# Differential cardiovascular responses to cutaneous afferent subtypes in a nociceptive intersegmental spinal reflex

**DOI:** 10.1038/s41598-019-54072-7

**Published:** 2019-12-13

**Authors:** Hyun Joon Lee, Jason M. White, Jumi Chung, Patrick Malone, Stephen P. DeWeerth, Keith E. Tansey

**Affiliations:** 1 Departments of Neurology and Physiology, Emory University, Atlanta, GA USA; 2 Department of Biomedical Engineering, Georgia Institute of Technology/Emory University, Atlanta, GA USA; 3 School of Electrical and Computer Engineering, Georgia Institute of Technology, Atlanta, GA USA; 4 Spinal Cord Injury Clinic, Atlanta VA Medical Center, Atlanta, GA USA; 5 Departments of Neurology, University of Mississippi Medical Center, Jackson, MS USA; 6 Departments of Neurobiology and Anatomical Sciences, University of Mississippi Medical Center, Jackson, MS USA; 7 Departments of Neurosurgery, University of Mississippi Medical Center, Jackson, MS USA; 8 G.V. (Sonny) Montgomery VA Medical Center, Jackson, MS USA; 9 NeuroRobotics Lab, Methodist Rehabilitation Center, Jackson, MS USA

**Keywords:** Sensory processing, Neurophysiology

## Abstract

Electrical stimulation to segmental dorsal cutaneous nerves (DCNs) activates a nociceptive sensorimotor reflex and the same afferent stimulation also evokes blood pressure (BP) and heart rate (HR) responses in rats. To investigate the relationship between those cardiovascular responses and the activation of nociceptive afferents, we analyzed BP and HR responses to electrical stimulations at each DCN from T6 to L1 at 0.5 mA to activate A-fiber alone or 5 mA to activate both A- and C-fibers at different frequencies. Evoked cardiovascular responses showed a decrease and then an increase in BP and an increase and then a plateau in HR. Segmentally, both cardiovascular responses tended to be larger when evoked from the more rostral DCNs. Stimulation frequency had a larger effect on cardiovascular responses than the rostrocaudal level of the DCN input. Stimulation strength showed a large effect on BP changes dependent on C-fibers whereas HR changes were dependent on A-fibers. Additional A-fiber activation by stimulating up to 4 adjacent DCNs concurrently, but only at 0.5 mA, affected HR but not BP. These data support that cutaneous nociceptive afferent subtypes preferentially contribute to different cardiovascular responses, A-fibers to HR and C-fibers to BP, with temporal (stimulation frequency) and spatial (rostrocaudal level) dynamics.

## Introduction

The relationship between pain and a closely following autonomic response is well established^1–4^. Activation of visceral^5^, muscular^6^, and cutaneous^7^ pain reflexes all produce measurable autonomic responses. More recently, it has even been proposed that the nociceptive and autonomic systems are two parts of an interoceptive system^8,9^. Autonomic responses to nociceptive input encompass a wide range of behaviors, including bladder function^10^, gastric motility^11^, and cardiovascular responses. Aside from the well-known examples of the importance of blood pressure (BP) in many pathologies e.g. cardiovascular disease, BP is also important in its relationship with nociception, such as in autonomic dysreflexia where a noxious sensory input below the severe high thoracic or cervical spinal cord injury can cause life threatening, episodic increases in BP^12,13^.

The effects of noxious stimuli on BP and heart rate (HR) have been compared by natural and electrical stimuli^4,14^, muscle/cutaneous/visceral afferents^5,15^, stimulation frequencies^4^, stimulation strengths^16^, anesthetic conditions^5^, body temperatures^17^, and spinal-segment specific activation^18,19^. Although these previous studies have extensively investigated those stimulation parameters separately, their interactions are not clear, nor do we know the comparative efficacy in producing cardiovascular responses among those stimulation parameters. For instance, in spinal cord injury, a better physiological understanding what nociceptive afferent subtypes, how much, and activated in what frequency are necessary to produce autonomic dysreflexia would be interesting in addition to important clinical questions related to the segmental differences (rostral or caudal to the level of injury).

Noxious input can concurrently evoke motor and autonomic responses. The spinal neural circuits for both nociception and the autonomic nervous system share input from the periaqueductal grey via the rostral ventrolateral medulla in the brainstem^20,21^, and the two systems are integrated at the brainstem level^22^. However, the close relationship between nociceptive afferent activation and autonomic regulations represent a gap in our current understanding of neurophysiology.

**Figure 1:**
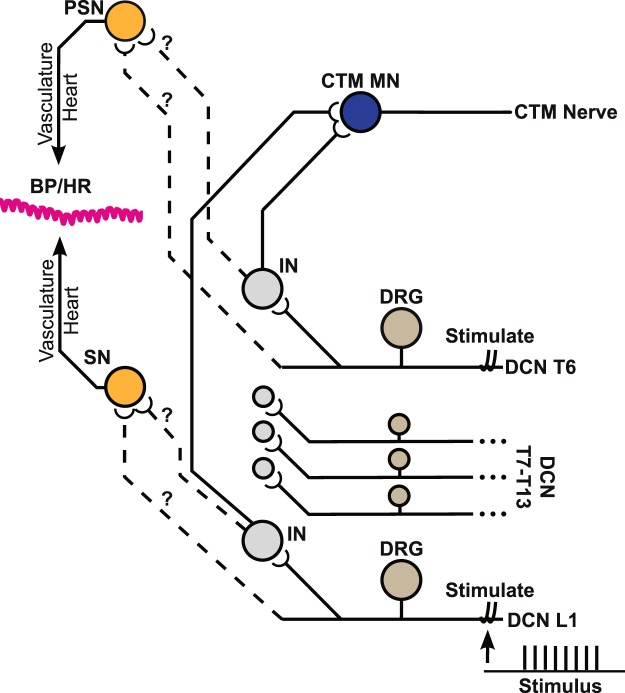
Cutaneus trunci muscle reflex and nociceptive-autonomic neural connection diagram. We included both known (solid lines) and unknown (dashed lines) but implied connections. We anticipate similar connections may exist between both rostral and caudal DCNs to both parts of the autonomic nervous system, showing the rostral DCNs only connected to the parasympathetic neurons is only for simplicity in the diagram. DCN: Dorsal Cutaneous Nerve, DRG: Dorsal Root Ganglion, IN: (spinal) InterNeuron, SN: Sympathetic Neuron, PSN: Parasympathetic Neuron, CTM: Cutaneus Trunci Muscle, MN: MotoNeuron, BP: Blood Pressure, and HR: Heart Rate.

The cutaneus trunci muscle (CTM) reflex^23–28^ provides a good model system to study both nociceptive-motor reflexes and nociceptive-cardiovascular responses from the same noxious stimuli. The CTM reflex is a relatively simple intersegmental cutaneous nociceptive reflex circuit in non-primate mammals which has been used to study functional recovery of cross-injury circuits in animal spinal cord injury models of the guinea pig^29,30^. In larger mammals, like dogs and horses, the CTM reflex is called the panniculus reflex^31^. The reflex is mediated by a polysynaptic circuit (Fig. 1), consisting of: (1) primary afferents in segmental dorsal cutaneous nerves (DCNs) from lumbar to thoracic levels, (2) propriospinal interneurons, and (3) the CTM motoneuron pool from C7 to T1^25^. The CTM reflex responses are known to be nociceptive specific in the rat generating two timely separate phases: an early phase supposed to be mediated by A-fibers (particularly Aδ) and a late phase mediated by C-fibers^24,27,32,33^. Previous studies were able to activate these afferent populations depending on stimulation strengths using a low stimulation strength for the early phase alone and a high stimulation strength for both early and late phases^24,26,28^. In our previous reports, the activation of DCNs at various stimulation frequencies at different spinal segmental levels demonstrated the temporal and spatial dynamics of the CTM reflex responses respectively^28^. DCN A- and C-fibers selectively labeled with axon-specific transganglionic tracers at different thoracic (T7 vs T13) levels also demonstrated that central projection patterns of those nociceptive fibers and their synaptic terminations contributed to the somatotopic (i.e. spinal segmental) organization of DCN-evoked CTM reflex responses^27,34^.

**Figure 2:**
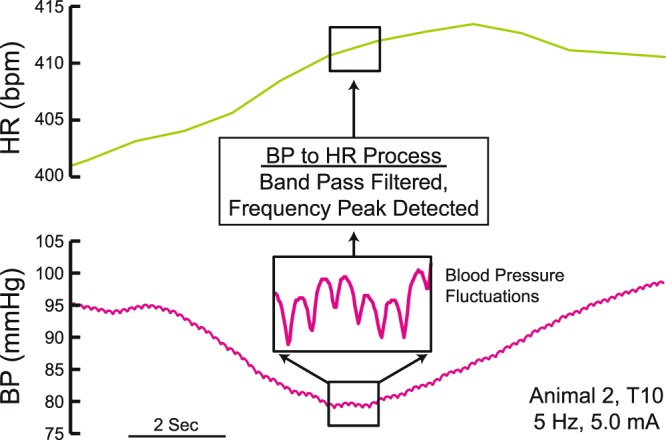
Processing of blood pressure trace to get heart rate. Representative blood pressure data shown at the bottom were previously filtered at 100 Hz. The small fluctuations in blood pressure trace are highlighted to show individual heartbeats. To calculate heart rate, the blood pressure was bandpass filtered between 3 and 12 Hz (180–720 beats per minute (bpm)), the power spectral density was calculated, and the peak was determined every second to compute heart rate, reported in bpm (top chart).

The activation of DCNs also evokes cardiovascular responses although the neurophysiological connections from cutaneous nociceptive afferents to the sympathetic and parasympathetic systems are not clearly identified (Fig. 1). Based on the temporal and spatial dynamics of the CTM reflex responses^24,26–28^, we hypothesized that the nociceptive-cardiovascular spinal reflex may be dependent on the dynamics of cutaneous afferent inputs in terms of stimulation strength (i.e. afferent subtypes), frequency, and spinal levels. In this study, BP responses were recorded in responses to DCN stimulations and HR responses were calculated from the pulse pressure fluctuations of BP response recordings in rats (Fig. 2). To understand the spatial and temporal dynamics of the nociceptive cardiovascular responses, we first analyzed BP and HR responses evoked by DCN stimulations at different spinal segmental levels at different frequencies. We also compared those cardiovascular responses evoked with single DCN stimulation at different stimulation strengths (A-fiber alone vs A- and C-fibers) or with multiple DCN stimulation only at A-fiber strength (additional recruitment of A-fiber alone from different DCNs) to investigate whether DCN afferent subtypes produce differential cardiovascular responses (BP vs HR).

## Results

### BP and HR Responses evoked by DCN stimulations

**Figure 3:**
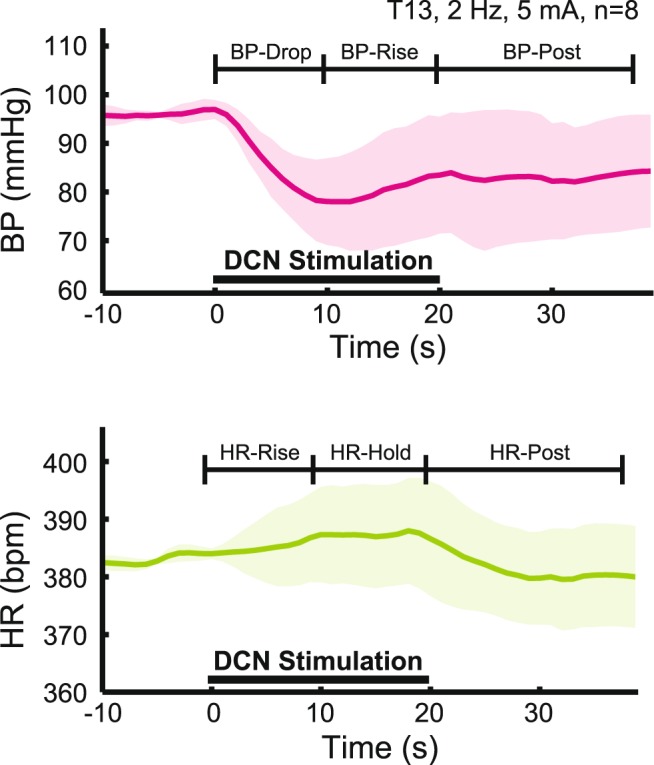
Typical Response to stimulation. The plots show the blood pressure (BP, top) and heart rate (HR, bottom) responses for T13 DCN stimulation at 2 Hz and 5 mA. Averaged responses across 8 animals were shown with solid lines, and the shaded area shown in the background is the standard deviation. The 20 seconds over which the DCN was stimulated is marked in the two panels as “DCN Stimulation.” The time-window for BP-Drop and HR-Rise was 0–9 seconds, for BP-Rise and HR-Hold was 9–20 seconds, and for BP-Post and HR-Post was 20–38 seconds.

The representative BP response (Fig. 3, upper panel) to DCN stimulation was an initial decrease (BP-Drop phase) followed by an increase (BP-Rise phase), recovering the resting BP level before the next stimulus train onset after 2 minutes. The minimum BP was reached between 7 and 12 seconds into the stimulus train and a simplified time window was set at 9 seconds for the purpose of analysis. We tracked the post-stimulation response out to 18 seconds (BP-Post phase).

The representative HR response (Fig. 3, lower panel) to DCN stimulation was an increase initially (HR-Rise phase), for the first 8–11 seconds (simplified to 9 seconds for the purpose of analysis), followed by a plateau until the end of stimulation (HR-Hold phase). HR dropped slightly below the baseline after the stimulation ended (HR-Post phase) and recovered before the next stimulation started. As with BP, we recorded the post-stimulation response for 18 seconds after stimulation ended.

### Stimulation strength

**Figure 4:**
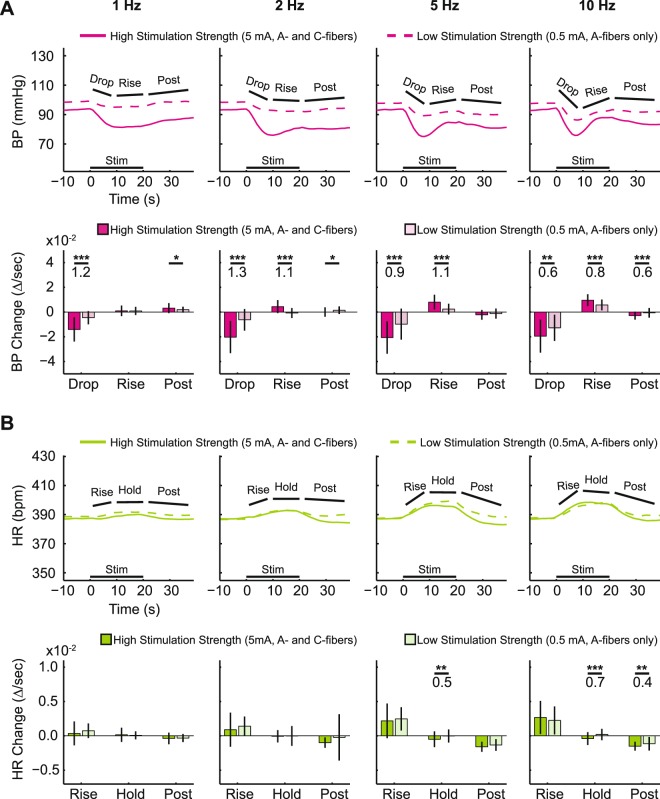
Relationship between cardiovascular responses at different stimulation strengths across different stimulation frequencies. In the top two rows of plots (**A**), blood pressure (BP, magenta) is shown, and in the bottom two rows (**B**), heart rate (HR, green) is shown. In the 1^st^ row (BP) and 3^rd^ row (HR), the time courses of the mean responses are plotted with the selected phases highlighted for both 5 mA (high-stimulation strength, solid line) and 0.5 mA (low stimulation strength, dashed line). The stimulation time is shown with the black bar on the bottom. In the 2^nd^ row (BP) and 4^th^ row (HR), a statistical comparison of the autonomic response changes per second are shown for the different response phases for both 5 mA (dark color) and 0.5 mA (light color). T-Tests were used to calculate that statistical significance between high and low stimulation strength evoked responses (*p < 0.05, **p < 0.01, ***p < 0.001). Cohen’s D was used to calculate effect size (shown under the *s). Each column shows a different stimulation frequency, with 1 Hz on the left, and 10 Hz on the right. For summarizing overall trends, all 8 animals and all 9 DCNs were combined for these plots.

Stimulation strength was a significant modulator of changes in BP (Fig. 4A), across all stimulation frequencies (1, 2, 5, or 10 Hz). The BP-Drop phase was much sharper and deeper in the high-stimulation-strength data (5 mA, A- and C-fiber activation) than in the low-stimulation-strength data (0.5 mA, A-fiber activation only), averaging 19% lower versus 8% lower respectively across frequencies, with a medium to very large effect size (0.6–1.3) between the two stimulation strengths. The BP-Rise phase was apparent in the high stimulation strength, but barely present in the low stimulation strength data, with large to very large effect sizes (0.8–1.1) at 2, 5, and 10 Hz stimulation frequencies. Only the 1 Hz stimulation data showed no significant difference in the BP-Rise phase between low- and high-stimulation-strength responses, despite their large difference in the BP-Drop phase of the signal. Qualitatively, the BP-Drop at 5 mA, 1 Hz and 0.5 mA, 10 Hz were similar suggesting the possible interaction of stimulation strength and frequency in determining the BP response profile.

The differences in HR response between high- and low-stimulation-strength were substantially less than the BP differences (Fig. 4B). The HR-response differences from stimulation strength did not reach significance at any stimulation frequency in the HR-Rise. In the HR-Hold, there were some significant differences at the higher stimulation frequencies (5 and 10 Hz), but those effect sizes were smaller than the effect sizes at the BP-Rise phase (0.5 for the HR-Hold phase vs 1.1 for the BP-Rise phase at 5 Hz). The HR-Post in Fig. 4B were only significant at 10 Hz stimulation frequency, with only a medium effect size (0.4). In summary, BP and HR respond very differently to stimulation-strength differences: BP is more sensitive to high-stimulation strength (A- and C-fiber activation), and HR is much less sensitive to high-stimulation strength (i.e. primarily sensitive to A-fiber activation).

### Stimulation frequency

**Figure 5:**
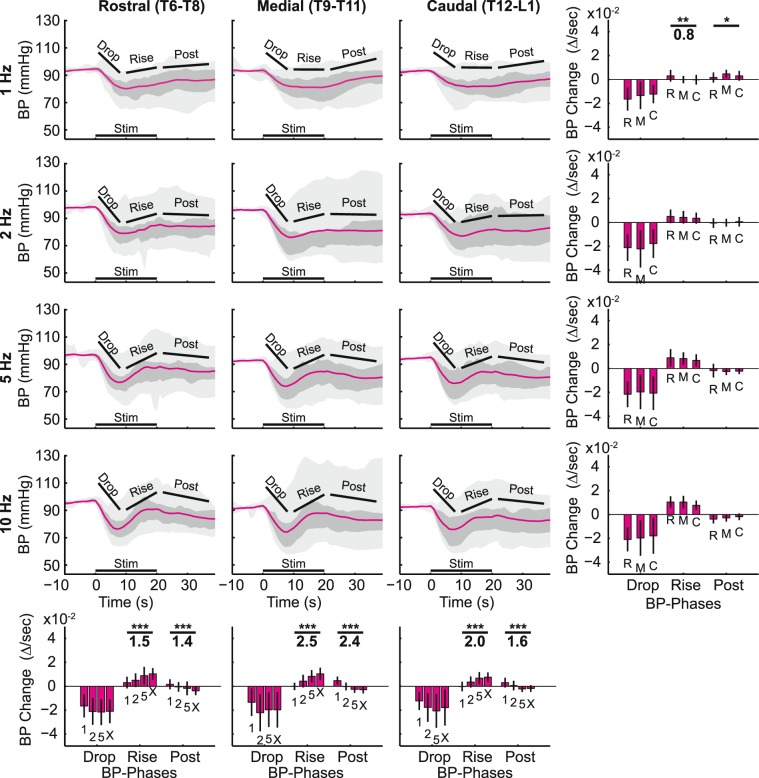
Blood pressure (BP) responses to stimulation of different DCNs at different stimulation frequencies. Traces show averaged (n = 24, 8 animals and 3 segments combined) BP data, all evoked at 5 mA stimulation strength. Stimulation of combined rostral (T6-T8), middle (T9-T11), and caudal (T12-L1) DCNs at all frequencies (1, 2, 5, and 10 Hz) are shown. On the plots, the magenta line represents the mean, the dark gray area represents the standard deviation above/below the mean, and the light gray area represents the range between minimal and maximal values at each time point in the response. The line at the bottom of the panels indicated DCN stimulation time (Stim). The BP data was summarized by the change per second in the response over the response phases described in the methods. Statistical analysis was performed along the panels on the right and bottom. The right panels compared responses from different DCN groups at different frequencies, and the bottom panels compared responses across stimulation frequencies at different DCN groups. In all cases, ANOVA was used to evaluate if the data came from the same statistical distribution (*p < 0.05, **p < 0.01, ***p < 0.001), and Cohen’s D was used to find the effect size for those with statistically significant differences of p < 0.01. Cohen’s D was calculated between the largest and smallest mean responses in those sets and is displayed under the p values. Error bars show standard deviation.

**Figure 6:**
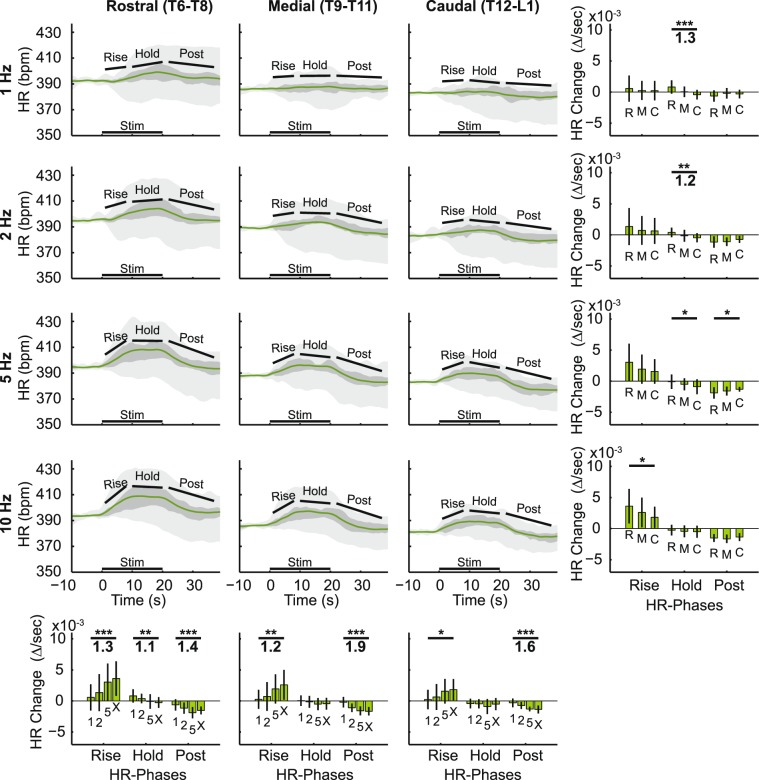
Heart rate (HR) responses to stimulation of different DCNs at different stimulation frequencies. Traces show averaged (n = 24, 8 animals, and 3 segments combined) HR data, all evoked at 5 mA stimulation strength. Stimulation of combined rostral (T6-T8), middle (T9-T11), and caudal (T12-L1) DCNs at all frequencies (1, 2, 5, and 10 Hz) are shown. On the plots, the green line represents the mean, the dark gray area represents the standard deviation above/below the mean, and the light gray area represents the range between minimal and maximal values at each time point in the response. The line at the bottom of the panels indicated DCN stimulation time (Stim). The HR data was summarized by the change per second in the response over the response phases described in the methods. Statistical analysis was performed along the panels on the right and bottom. The right panels compared responses from different DCN groups at different frequencies, and the bottom panels compared responses across stimulation frequencies at different DCN groups. In all cases, ANOVA was used to evaluate if the data came from the same statistical distribution (*p < 0.05, **p < 0.01, ***p < 0.001), and Cohen’s D was used to find the effect size for those with statistically significant differences of p < 0.01. Cohen’s D was calculated between the largest and smallest mean responses in those sets and is displayed under the p values. Error bars show standard deviation.

In Fig. 4, stimulation frequency modulated both the BP and HR responses, affecting the BP-Rise phase, and the HR-Rise phase more than the other phases in general. We further explored and quantified these stimulation frequency dependent differences at 5 mA stimulation in Fig. 5 for BP and Fig. 6 for HR. We stimulated at 9 different DCNs, sorting the cardiovascular responses into three groups depending on which spinal segmental levels they were evoked from: rostral (T6–T8), middle (T9–T11), and caudal (T12–L1) DCNs.

Qualitatively, at 1 Hz, the BP response just dropped and stayed low, but at higher frequencies (e.g. 10 Hz), the response involved a quick drop to the peak followed by an equally sharp rise (Figs. 4A and 5). These trends were quantified by comparing the BP response change as a slope (∆/second) at different stimulation frequencies as shown at the bottom of trace plots in Figs. 4A and 5. The effects of stimulation frequency on BP diverged, depending on which phase of the BP response was considered. Stimulation frequency was the more powerful modulator of the BP-Rise phase with larger effect sizes (1.5–2.5, bottom row in Fig. 5, ‘Rise’) than those from stimulation strength (0.8–1.1, Fig. 4A, ‘Rise’). On the other hand, the initial BP-Drop phase showed no significant stimulation-frequency-dependent trend. The BP-Post phase also showed significant stimulation-frequency-dependent differences with very large effect sizes (1.4–2.4, bottom row in Fig. 5). Overall, the size of the BP-Rise and BP-Post phases were most dependent on stimulation frequency.

HR changes were largely dependent on stimulation frequency, as can be seen from the different shapes of the HR responses (Figs. 4B and 6). The HR response from 1 Hz stimulation produced a nearly flat response, while 10 Hz stimulation produces a clear rise and plateau during stimulation, followed by a slow decline after the stimulus train ended. Statistically, the HR-Rise phase and HR-Post phase were most dependent on stimulation frequency (Fig. 6, bottom row) with very large effect sizes (1.2–1.9) as compared to only a few statistically significant difference from either the DCN being stimulated (Fig. 6, left column) or the stimulation strength (Fig. 4B, bottom row). The slope of the initial rise increased with the increasing stimulation frequency, peaking at 10 Hz with rostral DCN stimulation (Fig. 6, right bar graph in the bottom row). The HR-Post phase demonstrated large differences between different stimulation frequencies, with the HR reducing more at higher frequencies, loosely symmetrical with the frequency-dependent rise in HR.

### DCN (Spinal Segment)

There were generally less statistically significant, segmental differences in the cardiovascular responses (left columns in Figs. 5, 6) as compared to the differences in stimulation frequency (bottom rows in Figs. 5, 6) or strength (Fig. 4). In the cases that reached statistical significance, there were some rostrocaudal trends. There were more rostrocaudal differences in the HR response (Fig. 5) than in the BP response (Fig. 6). The HR-Hold phase showed the largest rostrocaudal trends: rostral DCN stimulation tended towards slight rises during the HR-Hold phase and caudal DCN stimulation tended towards slight drops. This rostrocaudal trend in the HR-Hold phase demonstrated very large effect sizes (1.2–1.3), as shown in the left column (Fig. 6). There was an additional, related trend where the HR increases in the earlier HR-Rise phase were larger with stimulation of the more rostral segments, but this trend only reached statistical significance in the 10 Hz data. Only the HR-Hold phase showed a strong DCN group dependence, as the other two HR phases (HR-Rise and HR-Post) were primarily dependent on the stimulation frequency.

Comparing across both the HR and BP responses, rostrocaudal trends in the responses were larger at low stimulation frequencies, with the only statistically significant change in BP found at 1 Hz stimulation (Fig. 5) and the largest effect sizes in the HR-Hold phase noted at 1 and 2 Hz (Fig. 6).

### Additional DCN A-fiber stimulation

**Figure 7:**
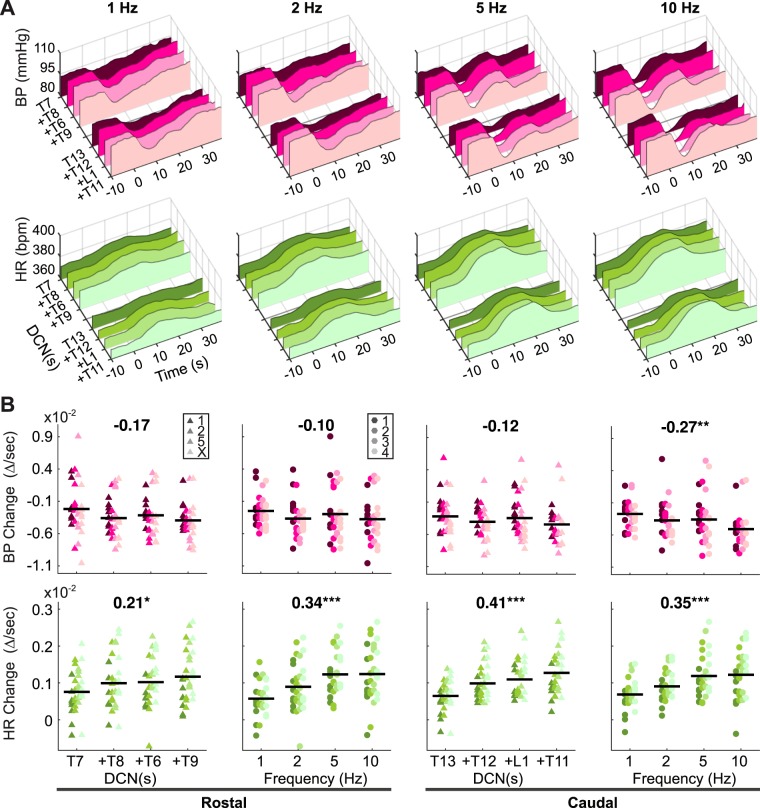
Blood pressure (BP) and Heart rate (HR) responses evoked by multiple DCN stimulation at A-fiber-dependent low stimulation strength (0.5 mA). BP and HR were averaged across animals (n = 8) and plotted on 3D graphs (**A**). From a single DCN at either a rostral (T7) or a caudal (T13) spinal level, adjacent DCNs were incrementally added one at a time up to 4 DCNs (added DCN is labeled with plus sign on the y-axis) and simultaneously stimulated at 0.5 mA, at different frequencies (1, 2, 5, and 10 Hz). Delta changes of BP and HR were calculated at the peak value over 38 seconds after the stimulation onset (**B**). Shown are averaged delta changes in each animal across stimulation frequencies at different DCN numbers stimulated (triangles) or across DCN numbers at different stimulation frequencies (circles), with their mean values across animals (n = 8, black lines). Symbol colors indicate different frequencies for triangles or different DCN numbers for circles. Correlation coefficients (Pearson’s r) and significance symbols (*p < 0.05, **p < 0.01, ***p < 0.001) were given at the top of each panel.

As BP and HR responses showed different sensitivity to stimulation strengths (Fig. 4A vs 4B), we further hypothesized that nociceptive DCN afferent subtypes, A- and C-fibers, contribute to different cardiovascular responses, HR vs BP respectively. To investigate this distinct contribution, A-fibers were activated at the low stimulation strength without C-fiber recruitment in the multisegmental DCNs. BP and HR responses were evoked while adjacent DCNs were added incrementally up to 4 DCNs starting at a rostral (T7) or a caudal (T13) level and stimulated simultaneously at 0.5 mA to activate additional A-fibers (Fig. 7A). For both BP and HR responses, multiple DCN stimulation did not change temporal patterns with 3 different time phases showing strong stimulation-frequency-dependence as seen with single DCN stimulation at low stimulation strength (Fig. 4). Qualitatively, the early time phases, BP-Drop and HR-Rise, were mostly affected by increasing DCN numbers, particularly HR-Drop, whereas there were no changes in later time phases.

To quantify effects of additional A-fiber activation on the cardiovascular responses, delta changes (∆/sec) were calculated at the peak values across all time phases (0–38 seconds, Fig. 7B). Correlation analysis was performed between delta changes across all stimulation frequencies and numbers of DCN stimulated at either rostral or caudal levels (plots with triangles). There was no significant correlation between BP changes and numbers of stimulated DCNs at both segmental levels. In contrast, HR changes gradually rose by increasing numbers of DCNs from 1 to 4 showing significant correlations at both rostral (r = 0.21) and caudal (r = 0.41) levels. This demonstrates that the activation of additional A-fibers with multiple DCN stimulation affects HR changes but not BP changes implying a selective contribution of nociceptive A-fibers to HR responses.

Correlation analysis was performed between delta changes across the multiple DCNs stimulated and stimulation frequencies (plots with circles, Fig. 7B). HR changes showed strong, positive correlations to stimulation frequency at both rostral (r = 0.34) and caudal (r = 0.35) levels. In comparison, BP changes were less dependent on stimulation frequencies showing only a significant correlation at caudal level (r = −0.27). The stimulation-frequency-dependence of BP tended to occur at high stimulation frequency e.g. 10 Hz. When values at 10 Hz were omitted in the correlation analysis, BP changes at the rostral level lost the significance (r = −0.09) whereas the correlation efficient for HR increased at both caudal (r = 0.42) and rostral (r = 0.41) levels. These data complement the interaction between stimulation strength and stimulation frequency reported in ‘Stimulation strength’ section.

### BP versus HR

**Figure 8:**
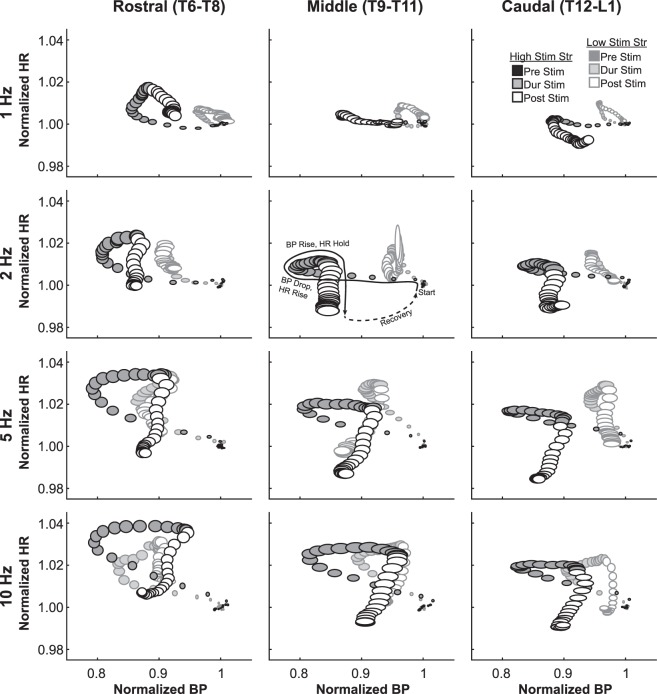
Heart rate (HR) versus blood pressure (BP) evoked by stimulation at different DCN groupings at different stimulation frequencies and strengths. There are 4 rows of plots, laid out in increasing frequency from top to bottom (1 Hz at top row, 10 Hz at bottom row). The 3 columns correspond to DCN groups: rostral (T6–T8), middle (T9–T11), and caudal (T12-L1). Within each plot, the values are normalized before averaging such that the starting value is 1 for both HR and BP. Low stimulation strength (0.5 mA) is shown partially grayed behind the high stimulation strength (5 mA) in each panel. In data from both stimulation strengths, the darkest ovals show movement in the HR versus BP space before stimulus began (“Pre Stim”). The medium gray ovals show the movement during stimulation (“Dur Stim”), and the white ovals show the recovery after stimulation ended (“Post Stim”). The size of the oval is 0.2x the standard deviation in each dimension.

In order to understand the relationship between BP and HR in a temporal manner, we traced the course of the cardiovascular responses at high stimulation strength (5 mA) over the time of DCN stimulation (circles with black outlines, Fig. 8). The HR vs BP space demonstrated several trends based on DCN segment and stimulation frequency. First, the drop in BP appeared to precede the rise in HR. BP would then rise and HR then fall, often slightly to below baseline levels. While many of the rostrocaudal changes in HR or BP were not as impressive when HR or BP were examined independently (Figs. 5, 6), differences in the overall qualitative paths of the BP-HR relationships were notable (Fig. 8). Rostral DCN stimulation tended to generate larger arcs in the BP-HR relationships than middle or caudal DCN stimulation, moving farther towards the high-heart-rate/low-blood-pressure corner in the top left in each panel in Fig. 8. In addition, another trend is the similarity in the evoked BP/HR path between rostral segmental activation at lower stimulation frequencies and caudal segmental activation at higher stimulation frequencies. This trend shows up by comparing diagonally from up-left to down-right (e.g. Rostral 5 Hz vs Middle 10 Hz and Middle 5 Hz vs Caudal 10 Hz), but these qualitative observations have not been evaluated statistically.

The cardiovascular responses evoked at low-stimulation-strength (circles with faded, gray outlines, Fig. 8) also showed a few trends when plotted in the HR vs BP space. Low-stimulation-strength (0.5 mA, A-fiber only) evoked HR responses in a similar range (traces in y-axis) as high-stimulation-strength (5 mA, A- and C-fiber) did. However, the range of BP responses (traces in x-axis) was much smaller at Low-stimulation-strength when compared to those ranges at high-stimulation-strength. The cardiovascular responses at low-stimulation-strength also showed a trend across different frequencies and DCN levels, producing an HR vs BP traces to a greater extent with higher stimulation frequencies at the more rostral segments. For instance, the Rostral DCN stimulation at 10 Hz (left lower corner) generated BP and HR ranges nearly as large as those ranges at the high-stimulation-strength stimulation at the same segment (Fig. 8).

## Discussion

We have studied autonomic responses to cutaneous nociceptive input in detail, exploring different features of the cardiovascular (BP and HR) responses, and whether they were correlated more with which DCN (spinal segment) was stimulated, what stimulus frequency was used, or which afferent population was activated (A-fiber alone at 0.5 mA vs A- and C-fiber at 5 mA). We have also utilized the additional recruitment of A-fibers by stimulating multiple DCNs at 0.5 mA to establish the selective contribution of nociceptive afferent subtypes to different cardiovascular responses: A-fibers to HR and C-fibers to BP.

The typical BP response to DCN stimulation was an initial decrease (BP-Drop phase), followed by a rise (BP-Rise phase), lasting through the end of the stimulation period. It is similar to what Koizumi and colleagues observed in cats stimulated at lumbar spinal nerves up to 10 Hz stimulation frequency^35^. After stimulation terminated (20 seconds), the BP generally dropped slightly (BP-Post phase), in proportion to the size of the rise. While a BP decrease in response to nociceptive input may seem counterintuitive, it is commonly seen in several cases: anesthetized animals^5,17^, unanesthetized animals with visceral nociceptive input^36,37^, and awake behaving animals experiencing certain types of cutaneous nociceptive input^14^. Following the early BP-Drop phase, BP increase (BP-Rise phase) was observed within the duration of stimulation trains, which might be a passive recovery from BP decrease or a temporally separate active response to noxious stimuli as reported previously^4,36^.

The typical HR response to DCN stimulation was an almost linear rise followed by a hold with insignificantly slight changes until the end of the 20-second pulse train, after which the HR fell back to the pre-stimulus value slowly. This response reflects the classic HR increase from cutaneous nociceptive input^4,36^. In fact, HR increases from nociceptive input are sufficiently common that one author has even proposed them as proxy measurement for pain in animal models^38^.

The cardiovascular responses to DCN stimulation show a strong dependence on stimulation strength, supporting a large role for which afferent population was activated (only A-fibers at 0.5 mA or A- and C-fibers at 5 mA). DCNs, like all other cutaneous nerves, contain large diameter myelinated A-α and A-β fibers (~30–80), smaller diameter myelinated A-δ fibers (~150–260), and unmyelinated C-fibers (~1500–2700), based on our lab’s previous anatomical quantification of those peripheral nerves^27^. These afferent fiber types are alternatively referred to as Group I/II fibers, Group III fibers, and Group IV fibers respectively. While DCNs have all subtypes of myelinated fibers, CTM reflex neurograms were not able to differentiate what proportion of which myelinated fiber population was stimulated due to how short the DCNs are in the rat^27,28^. As previous works have found, the rat CTM reflex was insensitive to the activation of low-threshold A-α or A-β fibers although it was sufficient to generate compound action potentials in DCNs^24,33^. Along similar lines, A-α/Group I muscle afferents have not been shown to produce any changes in BP or HR^16,39,40^. Therefore, both the CTM reflex and the BP/HR responses depend on activation of smaller diameter myelinated A-fibers and unmyelinated C-fibers.

Historically, systemic BP output to afferent stimulation has been controversial between depressor and pressor depending on types of nerve stimulated (muscle vs cutaneous), stimulation strengths (only A-fiber vs A- and C-fibers), and stimulation frequencies^4,16,39,41^. Despite of the controversy, these reports consistently demonstrated that primary afferent stimulation produced BP pressor only when both A- and C-fibers were activated at high stimulation frequency over 10 Hz which explains why we observed only depressor at given stimulation frequency up to 10 Hz. We found that moving from A-fiber-only stimulation (0.5 mA) to A- and C-fiber stimulation (5 mA) resulted in a significantly larger depressor response (Fig. 4A). This result might be due to an increased recruitment of smaller myelinated fibers leading to a larger depressor response as opposed to the addition of unmyelinated afferent activation. However, the activation of additional myelinated A-fibers in multiple DCNs at low stimulation strength was much less effective on the BP response changes than was the activation of both A- and C-fibers in a single DCN at high stimulation strength (Fig. 7 vs 4A). Although A-fibers alone can elicit small BP depressor, particularly in combination with higher frequencies, C-fibers play a major role in producing the early depressor responses in the cutaneous-autonomic system.

The late pressor response is less clear whether A- or C-fibers were actively involved in this response. It was also dependent on stimulation strength and stimulation frequency as was the early depressor. Based, however, on the symmetrical appearance of the late pressor following the early depressor mediated by A-fibers at 0.5 mA stimulation strength in single (Fig. 4A) and multiple (Fig. 7A) DCN stimulation, C-fibers were not required in the late pressor response. This remains only A-fibers as a candidate. As it has been known that C-fiber mediated responses were generally slower and longer than A-fiber mediated responses^35,41^, however, it is less convincible that A-fiber activation directly produced such a late response about 9 seconds after the stimulation train. Therefore, we speculate that the late BP increase is a result of either a passive recovery from the BP decrease, the early HR increase by means of A-fiber activation, or active neural process in a longer neural pathways e.g. the somato-sympathetic brainstem pathways.

In contrast to BP, HR response differences were nearly independent of stimulation strength (Fig. 4B), relying predominantly on the stimulation frequency at 5 mA stimulation (Fig. 6). As the A-fiber-only stimulation strength produced similar HR increases as A- and C-fiber activation, HR-Rise was mostly a result of A-fiber activation (Fig. 6). In addition, the activation of additional A-fibers in multiple DCN stimulation strongly correlated with the size of HR-Rise but not with BP-Drop (Fig. 7B). Taken all together, these data support the idea that A-fibers preferably contribute to HR and small BP depressor in stimulation-frequency-dependent manner while C-fibers exclusively contribute to BP depressor in normal animals. It might be relevant to study whether pathological autonomic reflexes, such as autonomic dysreflexia after spinal cord injury, show differential dependence on A-fiber and C-fiber sensory afferent inputs.

Stimulation frequency had generally the greatest effect among the stimulation parameters on both the BP-Rise and HR-Rise phases (Figs. 4, 5, 6). BP-Drop at 0.5 mA at 10 Hz stimulation reached the extent of which BP decreased at 5 mA at 1 Hz stimulation (Fig. 4). This suggests that A-fiber mediated BP-Drop requires relatively high stimulation frequency, at least, 10 Hz to make a sufficient BP change as C-fibers did. Indeed, BP responses at 0.5 mA significantly correlated to stimulation frequencies at the caudal level only when the data at 10 Hz stimulation was included (Fig. 7B). In addition, the stimulation of the maximal early BP-Drop phase at 5 mA was less consistent, sometimes observed at 2 Hz, and sometimes at 5 Hz, depending on the rostrocaudal location of the stimulated DCN (bottom panels, Fig. 5). These data demonstrated that A-fiber mediated BP-Drop was more stimulation-frequency-dependent than BP response mediated by C-fibers.

To the best of our knowledge, these results are the first to compare different stimulation frequencies of different nociceptive afferent populations by changing stimulation strength across multiple spinal segments evaluating both HR and BP responses. In addition, these results are the first to explore the rostrocaudal trends in these inputs across the thoracic and lumbar spinal cord. We found that stimulation of more rostral segments, closer to the cardiac sympathetic nerves, produced larger HR responses than stimulation of caudal segments (Fig. 6), while either level of input is roughly equivalent in terms of the BP response (Fig. 5).

Very little research has been published on segmental differences in the cardiovascular responses to nociception. It is known that BP and HR responses tend to be large from cutaneous stimulation in the fore or hind paw^19^, corresponding to inputs to the cervical and lumbar enlargements respectively. Similar results have been found in intact, anesthetized cats^18^. Our results have added a level of nuance by exploring the responses on a spinal-segment-by-segment basis, across the inter-enlargement thoracic spinal cord, through DCN stimulations.

BP and HR are regulated within certain ranges by the autonomic nervous system through various reflexes where a change in one can affect a change in the other. Our data included both HR changes and BP changes, so it is natural to question whether the causation is nociception→ BP→ HR, nociception→ HR→ BP, or nociception→ BP/nociception→ HR (independent of one another). From the perspective of time delays, the initial BP-Drop phase in the study happened before the HR increase, as can be seen in Fig. 8, which makes nociception→ HR→ BP unlikely. The parasympathetic BP→ HR baroreflex is known to be substantially attenuated by cutaneous nociception^42^. HR responses from various forms of cutaneous nociceptive stimulation were insensitive to whether the vagus nerve was intact or not^17,18^. However, there is also a purely sympathetic BP→ HR baroreflex^43^, which is still intact. Therefore, while we find it to be a less likely explanation, we cannot entirely dispute a BP→ HR interaction.

The results showed a divergence between early HR and BP responses in their activation and sensitivity to the stimulation frequency, as HR-Rise was exclusively activated by A-fibers and sensitive to frequency, while BP drops were activated by A-fibers in lesser extent in stimulation-frequency-dependent manner or by C-fibers in greater extent, and sensitive to stimulation-frequency as well. This divergence in activation and sensitivity supports parallel nociception→ BP/nociception→ HR pathways during nociceptive input. Other researchers have also reported similar cases of divergence between changes in HR and BP^17,18^. Therefore, we propose that during the early stimulation, the parallel nociception→ BP/nociception→ HR pathway dominates. Our ongoing studies in rats with high thoracic transection spinal cord injury that disconnects the brainstem pathways would elucidate whether the sympathetic baroreflex is involved in HR responses evoked by the cutaneous nociception. In addition, we will also be able to investigate what changes (A- vs C-fibers, BP vs HR) are responsible for the development of autonomic dysreflexia after that injury.

## Methods

All animal procedures were conducted with the approval of the Emory University Institutional Animal Care and Use Committees. All experiments were performed in accordance with relevant guidelines and regulations.

### Surgery

Female Long Evans rats (225–250 g, n = 16, Charles River Laboratories, Wilmington, MA) were anesthetized with pentobarbital (50 mg/kg, Nembutal, Ovation Pharmaceuticals, Deerfield, IL) and kept on a warming pad until they showed no paw withdrawal reflex to pinch or eye blink. The anterior neck and the back were shaved, and body temperature was maintained at 37 °C using a warming pad regulated by a feedback controller with a rectal probe (Physitemp Instruments Inc., Clifton, NJ) during the rest of surgical and recording procedures. The back skin was incised along the midline from near the base of the skull to the iliac crest, and the left DCNs from L1 to T6 were isolated from underlying fascia and cut distally. The anterior neck skin was incised, and the right carotid artery was exposed and freed from surrounding tissues. Care was taken to avoid injury to the vagus nerve during surgery and while the right carotid artery was cannulated and connected to a BP transducer (Harvard Apparatus, Holliston, MA) using PE 10 tubing filled with heparinized saline (2% heparin). The rats were placed in a stereotaxic frame (Kopf Instruments Inc.) to maintain the position of the rat, PE tubing, and electrodes and all exposed tissues were bathed in warm mineral oil. A DCN was placed on a bipolar stimulating electrode at a time (single DCN stimulation, see below) or multiple DCNs were placed on up to 4 electrodes simultaneously (multiple DCN stimulation, see below). Pentobarbital supplement (10% of the initial dose) was given intraperitoneally to maintain anesthetic level as needed (approximately hourly), where need was judged by the reemergence of the flexor reflex to paw pinch or the eye blink reflex. To avoid clotting in the PE 10 tubing, as detected by dampened pressure waves, the tubing was flushed occasionally during rest between stimulation epochs.

### Single DCN stimulation

A single segmental DCN from L1 to T6 was placed on a bipolar electrode connected to a stimulus isolator (ISO-Flex, A.M.P.I., Jerusalem, Israel) driven by a pulse stimulator (Master-8, A.M.P.I., Jerusalem, Israel). Each DCN was stimulated at either 0.5 mA or 5 mA stimulation strength (n = 8 at each strength, totaling n = 16) with pulse widths of 250 µs at different frequencies (1, 2, 5, and 10 Hz) for 20 seconds (Fig. 1). Although the fixed stimulation train time yields different stimulation numbers (20, 40, 100, 200 stimulations by given frequencies respectively), this ensures activation of cardiovascular responses which show a relatively long onset-to-peak latency (generally 9 seconds for BP and HR, see below ‘Blood Pressure Recording and Heart Rate Calculation’ and Fig. 3). The separation of 5 mA and 0.5 mA animals was required due to the consistent duration of the protocol after the initial pentobarbital injection. Single DCNs were stimulated in a caudal-to-rostral order (L1–T6) completing all stimulation frequencies at each DCN in order from lowest frequency to highest frequency to reduce possible habituation effects before moving on to the next DCN. A stimulation epoch lasting 20 seconds was started every 2 minutes, allowing the animal to rest for a sufficient amount of time for the BP and HR to return to baseline values before the next stimulation epoch began. The stimulation amplitudes were chosen based on empirical testing of the nociceptive-motor responses with only the early (A-fiber-dependent) phase of the CTM reflex evident at 0.5 mA, while 5 mA was consistently capable of maximally eliciting both the early and the ate (C-fiber-dependent) phases of the CTM reflex. This distinction (i.e. early phase of the CTM reflex being A-fiber-dependent and the late phase being C-fiber-dependent) is supported by previous work on the reflex^24,26^. In general, 3 mA stimulation was sufficient to produce the full late phase of the CTM reflex, but we used 5 mA to make sure we recruited the entire population of C-fibers even in DCNs with more neurovascular bundle tissue. Higher stimulation strengths or longer stimulation pulse widths failed to generate any further increases in the maximum CTM response.

### Multiple DCN stimulation

An additional group of rats (n = 8) were subjected to the surgical procedure described above and used for multiple DCN stimulations. A single DCN at a caudal (T13) level was placed on a stimulating electrode and stimulated at different stimulation frequencies (1, 2, 5, and 10 Hz) for 20 seconds but only at 0.5 mA (A-fiber-dependent). An adjacent DCN branch was then placed on an additional electrode with a separate stimulus isolator and the stimulation trains were repeated simultaneously. Ultimately, up to 4 DCNs were added incrementally in the order of T13, T13 + T12, T13 + T12 + L1, and T13 + T12 + L1 + T11. This was repeated at a rostral level starting at T7 in the order of T7, T7 + T8, T7 + T8 + T6, and T7 + T8 + T6 + T9. Consistent with the single DCN stimulation protocol, all stimulation frequencies at each DCN combination were started every 2 minutes in the order from lowest frequency to highest frequency before adding the next DCN.

### Blood pressure recording and heart rate derivation

The BP was continuously recorded during the entire stimulation protocol on a data acquisition board (USB 6259 BNC, National Instruments, Austin, TX) at a 10,000 Hz sampling rate. The stimulation from the stimulator was also registered in the recording file to locate each stimulation. The continuous recording data was transformed and processed in Matlab (Mathworks, Natick, MA). The BP signal was minimally filtered with a 100 Hz low-pass Butterworth filter, then converted to millimeter of mercury (mmHg) and cut from 10 seconds before each stimulus train to 20 seconds after the end of each stimulus train totaling 50 seconds.

The HR signal was calculated in beat per minute (bpm) from the pulse pressure fluctuations in the BP signal. A Butterworth band-pass filter (3–12 Hz, 180–720 bpm) was applied to the BP data, then the power spectral density was calculated using the periodogram method in Matlab and sampled at 0.5 Hz. The output showed the HR changes over time from DCN stimulation (Fig. 2). An artifact rejection algorithm was then applied where the HR data with large (>10%) deviations from the local median value within 4 seconds (±2 seconds) were replaced with local 2^nd^-order polynomial fits to the data.

For the purposes of both qualitative inspection and statistics, the cardiovascular responses from single DCN stimulation were time-windowed into specific phases (Fig. 3). For both BP and HR, the signals were split into three phases, chosen based on the timing of qualitative differences in how the autonomic signals responded to the stimulus. The three phases: BP-Drop or HR-Rise, for 9 seconds from the stimulus train onset (0–9 seconds), BP-Rise or HR-Hold, for 11 seconds after the first 9 seconds to the end of the stimulus train (9–20 seconds), and BP-Post or HR-Post, for 18 seconds from the end of stimulation (20–38 seconds). Although we collected data for 20 seconds post stimulation train, the last time window included 18 seconds in order to allow the BP response to be filtered for artifacts (described above). In each of these phases, the averaged change per second (∆/sec) was calculated. For the comprehensive comparison of the cardiovascular responses from multiple DCN stimulation, the maximal change was calculated over the time period across all time windows (0–38 seconds).

To relate temporal traces of HR and BP in Fig. 8, BP and HR values that started at slightly different baseline levels in each animal were normalized by averaged value for 10 seconds before the stimulation onsets. This approach has the advantage of being robust against different animal-to-animal differences and plotting different group means across frequencies, levels, and stimulation strengths consistently at the same baseline (1, 1).

### Statistical comparisons

In cases where two groupings were compared, the Student T-Test was used to establish significance (Fig. 4). In groupings where more than two values were compared, one-way analysis of variance (ANOVA) was used to establish significance, setting up the test to determine if the entire group came from the same probability distribution (Figs. 5, 6). In addition to the significance, to get a better estimation of how big an effect was, Cohen’s D was used to measure effect sizes where results were significantly different at a confidence level of 99% or more. In cases with more than two conditions to compare, the Cohen’s D was calculated between the conditions with the largest and smallest mean value. Following standard interpretation for Cohen’s D^44^, a small effect is noted at >0.2, a medium effect at >0.5, and a large effect at >0.8. A Cohen’s D of >1.1 is a very large effect size. For multiple DCN stimulation, we focused the correlational analysis on changes of different cardiovascular responses, BP and HR, with the number of DCNs stimulated. The maximal changes across all phases were used in order to calculate correlation coefficients (Pearson’s Correlation) of BP and HR responses against either the number of DCNs or the stimulation frequency using “corrcoef” function (Matlab). Significance was indicated above the correlation coefficients. In all figures, error bars represent standard deviation. Significance was generally shown at either >95% (*), >99% (**) or >99.9% (***) of the groups being different.

## Data Availability

The datasets generated during and/or analyzed during the current study are available from the corresponding author on reasonable request.
